# Liver fibrosis in patients with chronic liver diseases: are laboratory tests useful to diagnosis?

**Published:** 2011-05-01

**Authors:** Salvatore Leonardi, Giovanna Vitaliti

**Affiliations:** 1 Department of Pediatrics, University of Catania, Catania, Italy

**Keywords:** Liver fibrosis, Diagnosis

Dear Editor,

The article published in Hepatitis Monthy on noninvasive assessment of liver fibrosis with the aspartate transaminasis to platelet ratio index (APRI) in patients with chronic liver disease by Yilmaz et al. [1], is interesting and very useful to permit a diagnosis and staging of fibrosis, although this index does not seem to have a high sensitivity and specificity. In recent years, efforts have been made to develop non-invasive predictive models that may correlate with stage of fibrosis. One of the first non-invasive predictive models for patients with chronic hepatitis C (CHC) was the Fibrotest, which includes α2-macroglobulin, haptoglobin, γ-glutamil-transferase (GGT), apolipoprotein A1 and total bilirubin. However, considerable expenses and use of uncommon parameters reduce their clinical applicability. A few years later, the Forns’ score (age, GGT, cholesterol, platelets and prothrombin) and the APRI index (AST and platelets) overcame these draw-backs by use of only standard laboratory tests in the development of their predictive models.

Subsequent models include the ELF-score, the Hepascore and the Fibrometer [2]. Thus, actually, the models of noninvasive predictors of liver fibrosis can be divided into two main groups: models only consisting of simple routine tests (the S index, Hui model, Forns’ score and APRI) and models including special tests such hyaluronic acid and serum α2-macroglobulin (the SLFG model, Fibrometer and Hepascore) [3].

Yilmaz Y et al., found that the APRI was significantly associated with fibrosis scores in patients with CHC and NAFLD, but not in those with CHB. Nevertheless, CHB is the most frequent infectious cause of chronic liver disease worldwide. More than 400 million people are chronically infected with HBV. The virus is responsible for more than 300,000 cases of liver cancer annually [4].

Thus, it is relevant to find more significant approaches to the diagnosis of fibrosis in CHB. Recently, Zhou K et al. [2], compared different non-invasive methods to assess liver fibrosis and found that the S index was not only significantly associated with fibrosis in CHB patients, but it also had the highest predictive value in assessing significant fibrosis, compared with other analyzed models. The S index is based on routine laboratory markers such as γ-glutamiltransferase, platelets count and albu min (GGT, PLT and ALB) that are readily available to most clinicians managing patients with chronic HBV infection, so that no additional tests are needed. Moreover, it seems that AST, one of the two parameters considered in the APRI, did not show a significant correlation with liver fibrosis staging of CHB patients, determining a lower sensibility of APRI than S index. In fact, they found that in predicting significant fibrosis in CHB patients the sensitivity was 81% for S index and 71 % for APRI, and in predicting advanced fibrosis the sensitivity of the two models increased up to 89% for the S index and 81% for APRI. For detecting liver fibrosis in CHC, CHB and NAFLD patients, authors reported that APRI has a sensitivity of 72.2% and a specificity of 62.4% for the diagnosis of fibrosis in CHC patients; 60% and 62.4%, respectively for the NAFLD group; and 55% and 75.4%, respectively for CHB patients.

These data state that physicians cannot make their diagnosis of liver fibrosis only based on APRI, and biopsy is necessary for the diagnosis and staging of fibrosis. Therefore, it would be interesting to investigate the potential usefulness of other non-invasive tests such as liver stiffness measurement (LSM), FIB-4 [5] and FORNS biochemistry [6] indices as well as transient elastography [7], that showed in literature better sensitivity and specificity. Although most of the non-invasive predictive models are not able to give the exact staging of fibrosis due to serious overlap among patients with different stages of fibrosis, they have sufficient accuracy in predicting significant fibrosis in various liver diseases [8][9]. Furthermore, the combination of diagnostic models and other non-invasive techniques can improve the performance to a higher level. The combined use of transient elastography and Fibro test to evaluate liver fibrosis could avoid a biopsy procedure in most patients with CHC [10]. Combination of Fibroscan and S index will also an effective way of managing CHB patients, especially in the followup of antiviral therapy.

The detection and staging of liver fibrosis is surely crucial for management of patients with chronic liver disease. At present, liver biopsy is the gold standard method for staging fibrosis, but biopsies are poorly tolerated because they are invasive and associated with some discomfort and complications. In addition, limitations of biopsy include intra- and inter-observer variation and sampling error [2]. For all these reasons, we agree that it is necessary to find out new, reliable, and non-invasive diagnostic methods for identifying patients with liver fibrosis. Nevertheless, before implementing a model into practice, priority should be given to large scale validation studies.
